# Journey of a patient with chronic thromboembolic pulmonary hypertension

**DOI:** 10.1186/s40001-015-0112-x

**Published:** 2015-03-02

**Authors:** Dan Liu, Kai Hu, Heinz-Theo Pelzer, Stefan Störk, Frank Weidemann

**Affiliations:** Department of Internal Medicine I - Cardiology, University of Würzburg, Würzburg, Germany; Comprehensive Heart Failure Center, University of Würzburg, Würzburg, Germany; Department of Internal Medicine I - Pneumology, University of Würzburg, Würzburg, Germany; Medical Clinic II, Katharinen-Hospital Unna, Obere Husemannstraße 2, 59423 Unna, Germany

**Keywords:** Chronic thromboembolic pulmonary hypertension, Tricuspid pressure gradient

## Abstract

Right ventricle (RV) dysfunction is a key outcome determinant and a leading cause of death for patients with chronic thromboembolic pulmonary hypertension (CTEPH). In this report, we followed the 5-year clinical journey of a patient with CTEPH. The tricuspid pressure gradient was significantly increased in the early phase of CTEPH and “normalized” at the late phase of this patient’s clinical journey, but this “normalized” gradient is not a positive treatment response but rather an ominous sign of advancing right heart failure owing to an exhaustion of RV contractile function. Thus, appropriate interpretation of the tricuspid pressure gradient change is of importance for assessing RV dysfunction and treatment outcome during follow-up in patients with CTEPH. Besides systolic pulmonary artery pressure (SPAP), other RV functional parameters such as tricuspid annular plane systolic excursion, RV fractional area change, and RV longitudinal strain, together with clinical markers, may provide additional guidance regarding functional improvement or progression in patients with CTEPH.

## Background

Pulmonary thromboembolism serves as a common cause of pulmonary hypertension (PH), termed chronic thromboembolic pulmonary hypertension (CTEPH; WHO Group IV) [1,2]. Fibrous organization of persistent pulmonary emboli leads to increased pulmonary vascular resistance and promotes adverse pulmonary vascular remodeling, thus contributing to further rises of pulmonary artery pressure. This vicious cycle governs the clinical course of human CTEPH including right ventricular (RV) hypertrophy and, ultimately, progressive right heart failure. It is known that RV dysfunction is a key determinant of patient outcome and the leading cause of death for patients with CTEPH [3-5]. A thorough clinical work-up is required to establish the diagnosis of CTEPH and guide patients through either surgical and/or medical treatment. Echocardiography is widely used to screen individuals for suspected PH and routinely applied during clinical follow-up settings. Here, we report the 5-year clinical journey of a patient with CTEPH.

## Case presentation

**Figure 1:**
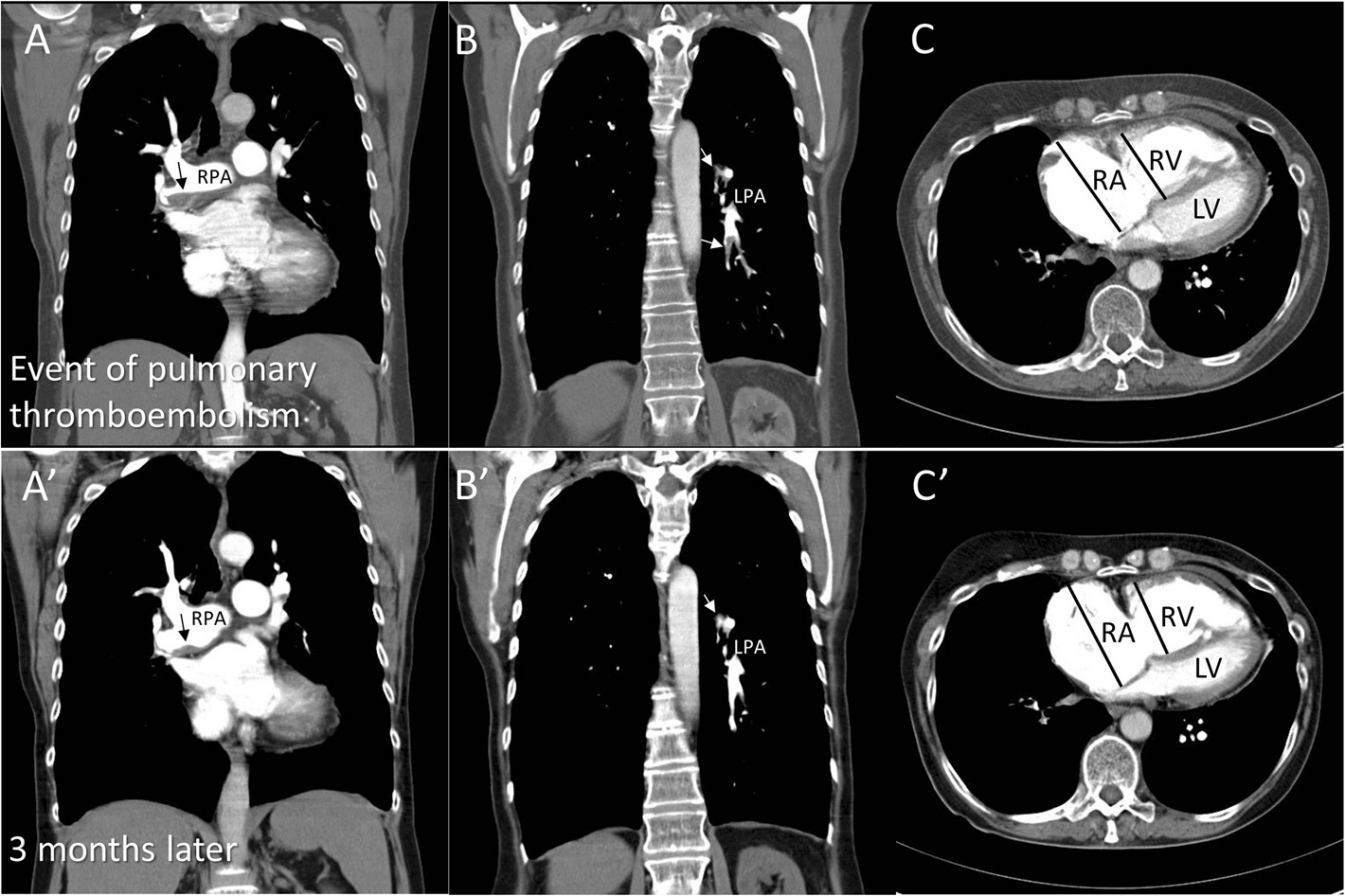
**Computed tomography scans at first clinical presentation and 3 months later. (A)** Coronal contrast-enhanced CT scan at first clinical presentation shows a flattened eccentric thrombus in the main right pulmonary artery (black arrows). **(A’)** Repeat CT scan after 3 months of treatment shows the shrunken thrombus formation (black arrow). **(B, B’)** Coronal contrast-enhanced CT scans at the time of first diagnosis and 3 months later show multiple filling defects both at the bifurcation and in the left pulmonary arteries (white arrows). **(C, C’)** Axial contrast-enhanced CT scans show dilatation of the right ventricle (RV), dilatation of the right atrium, and thickening of the RV free wall both at initial presentation and 3 months later. LPA: left pulmonary artery; LV: left ventricle; RA: right atrium; RPA: right pulmonary artery; RV: right ventricle.

**Figure 2:**
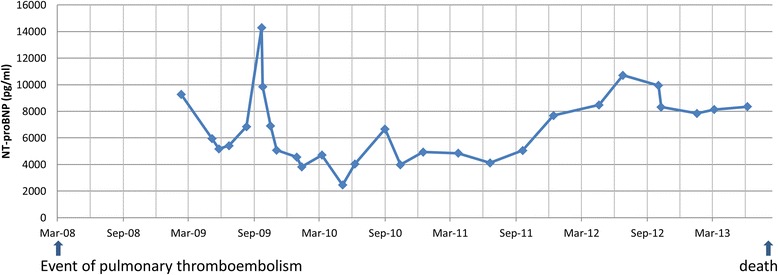
**Temporal changes of NT** **-** **proBNP measurements during follow** **-** **up.**

A woman aged 68 years was admitted to our hospital due to progressive dyspnea in March 2008. She presented mild bilateral leg edema at physical examination. The ECG showed acute right ventricular strain (SI/QIII) and sinus tachycardia (heart rate = 120 beats/min). Blood tests evidenced elevated serum D-dimer (2.59 mg/L, that is, fivefold increase). Computed tomography (CT) revealed extensive bilateral thrombi in proximal and peripheral pulmonary arteries plus RV dilation and hypertrophy (Figure 1A,B,C). Pulmonary function tests indicated GOLD II chronic obstructive pulmonary disease (COPD). Arterial blood gas analysis suggested hypocapnia and hypoxemia (pCO_2_ 30 mmHg; PO_2_ 64 mmHg; O_2_ saturation 94%). Initial transthoracic echocardiography (TTE; Table 1) showed a significantly dilated RV with D-shaped left ventricle (LV) and a dilated right atrium (RA). LV and left atrium dimensions as well as LV ejection fraction were normal. Doppler-derived maximal systolic pulmonary artery pressure (SPAP) was 69 mmHg (maximum trans-tricuspid pressure gradient of 59 mmHg + estimated RA pressure of 10 mmHg). Diagnosis of CTEPH was established by above examinations and right heart catheterization examination. Other causes of PH were excluded. Initial therapy consisted of sildenafil (3 × 20 mg/day), oral anticoagulation, and diuretics. Therapy was sequentially escalated towards triple therapy including inhaled iloprost (6 × 2.5 μg/day) and endothelin receptor blockade with ambrisentan (1 × 10 mg/day). The patient repeatedly refused evaluation for pulmonary endarterectomy (PEA) and remained clinically stable on WHO level III for 4 years. The N-terminal of the prohormone brain natriuretic peptide (NT-proBNP) levels also remained stable initially, but increased during long-term follow-up (from 2,400 to 14,300 pg/mL; Figure 2). The patient presented with decompensated right heart failure in October 2012 and was evaluated for PEA and heart/lung transplantation; however, the evaluation results were negative. After a stable period of several months under standard medication, the patient presented again with decompensated right heart failure in August 2013 and died in hospital due to cardiogenic shock.

**Table 1:** **Echocardiographic**, **right heart catheterization and arterial blood gas analysis characteristics**

	**Oct** **-** **07**	**Mar** **-** **08 initiation**	**Apr** **-** **09**	**Apr** **-** **12**	**Oct** **-** **12**	**Aug** **-** **13**
*Echocardiography*						
LVEDD (mm)	39	38	39	37	39	39
IVSd (mm)	8	8	8	8	8	8
CI (L/min,Teich)	3.16	2.71	2.46	2.31	1.61	1.86
LAD (mm)	27	29	28	28	32	30
RVD (mm)	37	46	48	51	53	48
RVd (mm)	6	7	7	7	7	7
RAA (cm^2^)	13	23	26	30	30	20
LV EF (%)	69	69	68	85	73	72
RV FAC (%)	46	24	20	18	18	21
MAPSE_septal (mm)	9	6	5	7	7	8
MAPSE_lateral (mm)	10	8	8	9	9	10
TAPSE (mm)	18	15	13	13	12	13
LV diastolic function	abnormal relaxation	abnormal relaxation	-	abnormal relaxation	-	abnormal relaxation
TR maxV (m/s)	2.5	3.8	3.8	4.2	3.0	2.2
TR maxPG (mmHg)	25	59	58	71	36	19
VTI_RVOT_ (cm)	-	-	9.1	12.0	-	10.7
Estimated PVR (Woods units)	-	-	4.02	3.34	-	1.90
RAP (mmHg)	5	10	15	15	15	10
SPAP (mmHg)	30	69	73	86	51	29
RVOT diameter (mm)	-	37	39	41	-	40
Main PA diameter (mm)	-	24	24	25	-	30
Pericardial effusion (mm)	0	5	3	0	4	3
Pleural effusion	-	yes	-	-	yes	-
*Right heart catheterization*						
PA mean pressure (mmHg)	-	38	42	-	45	-
PA systolic pressure (mmHg)	-	71	75	-	81	-
RA mean pressure (mmHg)	-	4	6	-	14	-
RV systolic pressure (mmHg)	-	67	75	-	84	-
PA O_2_ (%)	-	47	53	-	51	-
*Arterial blood gas analysis*						
PO_2_ (mmHg)	-	64	56	59	50	58
PCO_2_ (mmHg)	-	30	33	35	43	41
O_2_ saturation (%)	-	94	91	93	89	93
*6MWD (m)*	*-*	*-*	*200*	*190*	*-*	*200*

Initiation represents the first hospitalization of this patient in our hospital because of progressive dyspnea. LVEDD: end-diastolic left ventricular dimension; IVSd: end-diastolic interventricular septal thickness; LAD: end-systolic left atrial diameter; RVD: end-diastolic basal right ventricular dimension; RVd: end-diastolic right ventricular free wall thickness; RAA: end-systolic right atrial area; LV: left ventricle; EF: ejection fraction; RV: right ventricle; FAC: fractional area change; MAPSE: mitral annular plane systolic excursion; TAPSE: tricuspid annular plane systolic excursion; TR: tricuspid regurgitation; maxV: maximal velocity; maxPG: maximal pressure gradient; RVOT: right ventricular outflow tract; VTI: velocity-time integral; PVR: pulmonary vascular resistance, estimated PVR by echocardiography formula: 10 × (TR maxV/VTI_RVOT_) − 0.16; CI: cardiac output indexed to body surface area; Woods units: mmHg⋅min/L; RAP: estimated right atrial pressure; PA: pulmonary artery; RA: right atrium; 6MWD: 6-min walk distance test.

**Figure 3:**
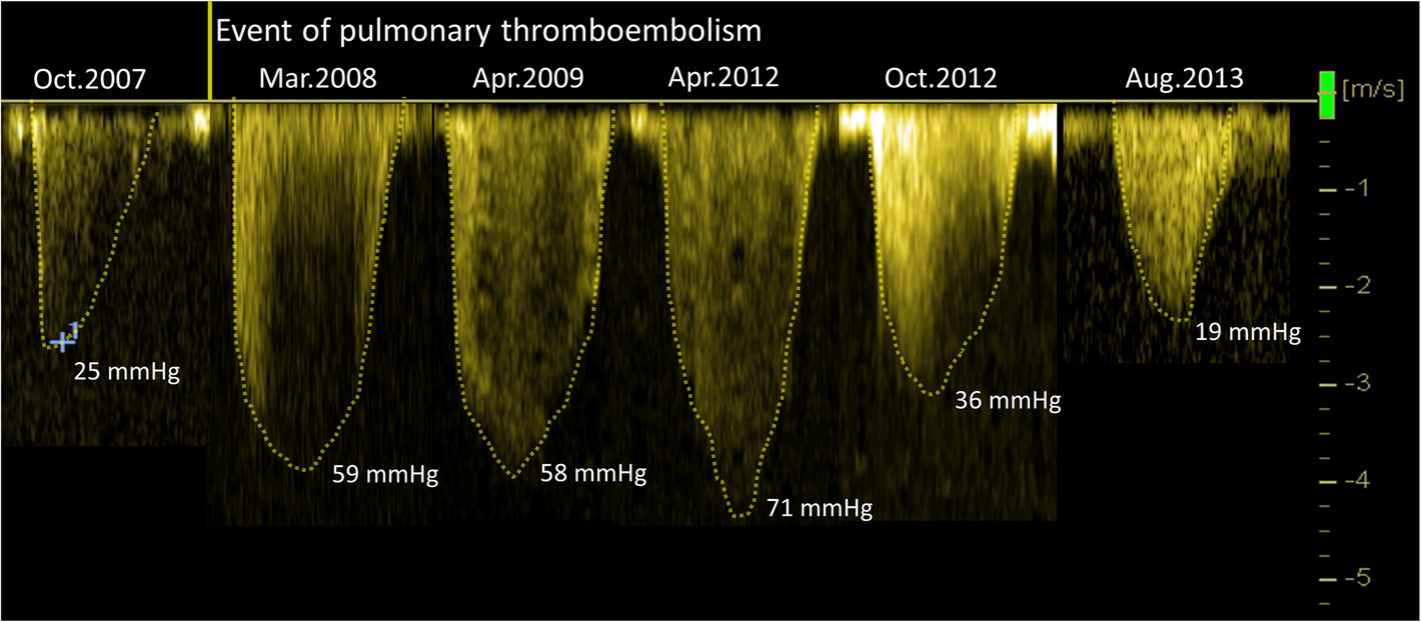
**Serial echocardiographic observations of tricuspid regurgitant jet (continuous**-**wave Doppler recording).** Note the pseudo-normal trans-tricuspid pressure gradient (19 mmHg) with the pathological late-peaking velocity spectrum at the final examination (August 2013), clearly distinct from the normal pressure gradient exhibiting an early-peaking velocity spectrum at the examination in 2007, that is, prior to the clinical event of pulmonary thromboembolism.

**Figure 4:**
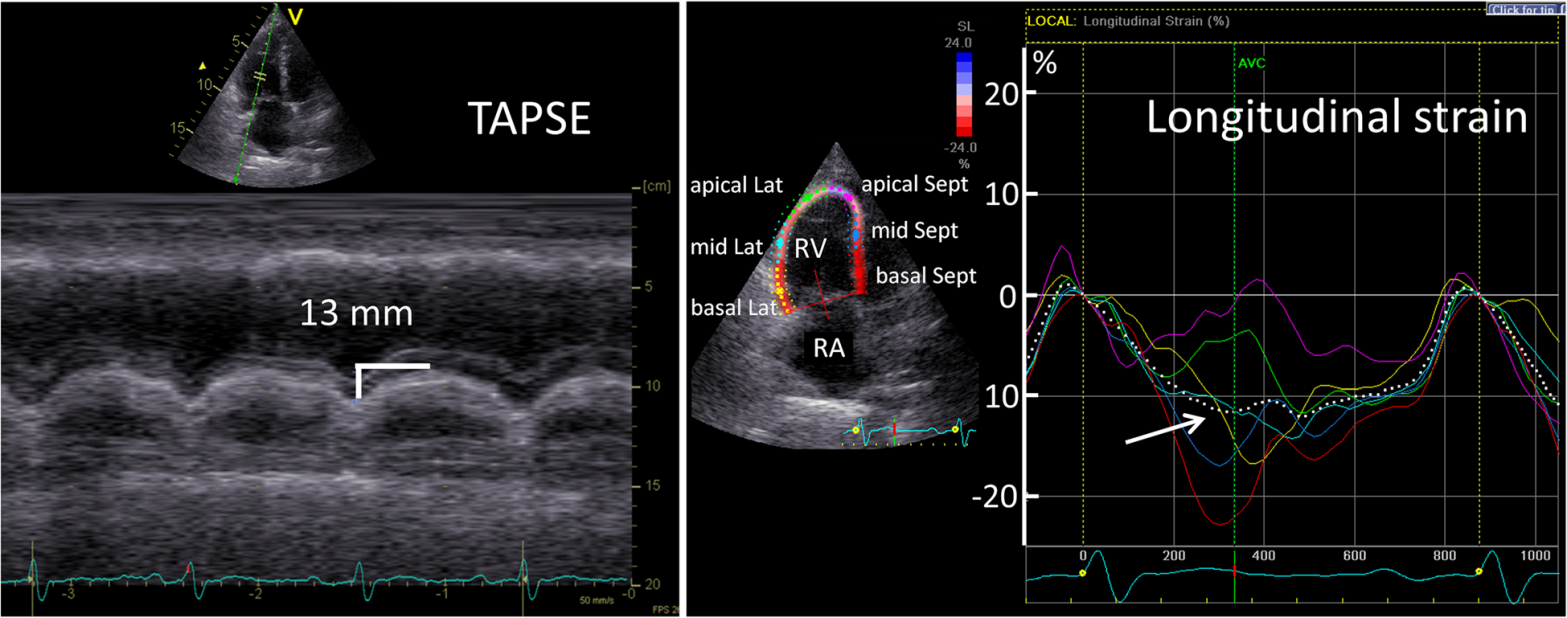
**Measurements of tricuspid annular plane systolic excursion (TAPSE**; **left) and right ventricular longitudinal systolic strain (LSsys**; **right) derived from two**-**dimensional speckle tracking imaging at the final examination (August 2013).** Solid-colored lines indicate corresponding segmental strain curves and white dashed line indicates global strain curve on the right panel. Note the considerably reduced measurement values for TAPSE (normal range: >20 mm) and right ventricular global LSsys (−11%). Lat: RV lateral wall; RA: right atrium; RV: right ventricle; Sept: interventricular septum.

Serial TTE (Figure 3) revealed a constant elevation of Doppler-derived SPAP with the highest value of 86 mmHg between March 2008 and April 2012. Right heart catheterization showed correspondingly high mean pulmonary arterial pressure of 38 to 45 mmHg at this period (Table 1). Of note, Doppler-derived SPAP gradually decreased over the last two examinations. Reduced tricuspid annular plane systolic excursion (TAPSE; Figure 4, left) and RV fractional area (FAC) were additionally seen throughout the follow-up. Two-dimensional speckle tracking imaging (STI) revealed severely impaired RV longitudinal function (global longitudinal strain = −11%) with predominantly compromised function at the apex (Figure 3, right). Additionally, tricuspid regurgitation (TR) continuous-wave Doppler demonstrated a nominally “normal” peak pressure gradient (19 mmHg) with a late peaking velocity spectrum at the final examination. Interestingly, a normal tricuspid pressure gradient with a normal early peaking velocity spectrum was shown by Doppler echocardiography performed in 2007 before the clinical event of pulmonary thromboembolism (Figure 3).

NT-proBNP levels, 6-min walk distance (6MWD), and invasive hemodynamic monitoring suggested that severe PH existed during the whole disease journey. Extremely enlarged RV and RA, dilated main PA diameter, and decreased cardiac output, as well as increased pulmonary vascular resistance also provided circumstantial evidence on progressive worsening in both RV volume and pressure overload in this patient. However, significantly elevated tricuspid pressure gradient in the early phase of CTEPH normalized at the late phase of this patient’s clinical journey. The potential reasons for pseudo-normalization of SPAP during development in this patient remain an issue of speculation. This decline of the pressure gradient is not due to improvement of tricuspid regurgitation but rather a feature of progressive right ventricular dysfunction, as indicated by a sustained reduction in TAPSE, RV FAC, and correspondingly reduced RV longitudinal strain in STI (Figure 3). Previous experimental study on chronic RV pressure overload model proved that RV systolic function is preserved, but diastolic function is impaired. To compensate, RA contractility increases to maintain filling stiffened ventricle, and the atrium acts as a reservoir than a conduit [6]. As time passes, RA pressure comes to be further increased due to the exhaustion of RA contractility. When the increased degree in the RA pressure is more than in the RV pressure, the pressure difference between RV and RA (tricuspid pressure gradient) will be reduced. Thus, we speculate that pseudo-normalization of the pressure gradient should not be interpreted as a positive treatment response but rather as an ominous sign of advancing right heart failure owing to an exhaustion of RV and RA contractile function. The shape of the velocity spectrum is another important sign in this context as the late-peaking velocity trace might be related to RV contractile dysfunction.

Additionally, we observed the discrepancy between echocardiographic-derived SPAP (reduced) and right heart catheterization measured pulmonary artery systolic pressure (increased) during the late course of PH in this patient. The underlying reason might be that echocardiographic-derived SPAP reflects the pressure gradient between RV and RA, which is low due to increased RA pressure in this patient at late disease phase. While invasive right heart catheterization-measured SPAP presents the real increased pressure situation at pulmonary artery.

In comparison with tricuspid pressure gradient, some clinical indices and other echocardiographic parameters might be additionally useful for predicting the outcome in this case. When retrospectively reviewing the full journey of this patient, sustained elevated NT-proBNP levels and a significantly low 6MWD were observed. Previous studies showed that BNP and NT-proBNP correlated well with RV function and prognosis in several forms of pulmonary hypertension [7-9]. The 6MWD has been widely used for measuring the response to therapeutic interventions for pulmonary and cardiac disease [8]. Our observation also demonstrated the value of monitoring 6MWD for evaluating PH disease progression. Therefore, monitoring these clinical indices during follow-up might be helpful for predicting the adverse outcome in patients with CTEPH besides standard echocardiographic parameters including SPAP, TAPSE, RV FAC, and STI.

## Conclusions

In conclusion, “normalized” echocardiography-derived SPAP with the progression of the disease is a feature of progressive right heart dysfunction in patients with CTEPH. Besides SPAP, other RV functional parameters such as TAPSE, RV FAC, and RV longitudinal strain, together with clinical markers, may provide additionally useful guidance regarding functional improvement or progression in patients with CTEPH.

## Consent

The study was approved by Local Ethics Committee at the University of Würzburg and conducted in accordance to the Declaration of Helsinki.

Written informed consent was obtained from the patient’s guardian for publication of this case report and any accompanying images. A copy of the written consent is available for review by the Editor-in-Chief of this journal.
